# Impact of ankylosing spondylitis on depression: a nationwide cohort study

**DOI:** 10.1038/s41598-019-43155-0

**Published:** 2019-05-01

**Authors:** Jin-Sung Park, Hae-Dong Jang, Jae-Young Hong, Ye-Soo Park, Kyungdo Han, Seung-Woo Suh, Si-Yong Park, Bo-Taek Kim

**Affiliations:** 1 Department of Orthopedics, Korea University Ansan Hospital, Ansan, South Korea; 2 Department of Orthopedics, Soonchunhyang University Bucheon Hospital, Bucheon, South Korea; 3 Department of Orthopedics, Hanyang University Guri Hospital, Guri, South Korea; 4 Department of Biostatistics, Biomedicine & Health Sciences, Catholic University, Seoul, South Korea; 5 Scoliosis Research Institute, Department of Orthopedics, Korea University Guro Hospital, Seoul, South Korea; 6 Department of Orthopedics, Korea University Anam Hospital, Seoul, South Korea

**Keywords:** Ankylosing spondylitis, Epidemiology

## Abstract

The aim of this study is to determine the relationship between AS and subsequent depression. This study was conducted using a nationwide dataset available in Korean National Health Insurance System (KNHIS). We identified 11,465 newly diagnosed AS patients and 57,325 patients without AS in the ratio of 1:5 matched by sex, age, and index date, between 2010 and 2014. We investigated any latent characteristics in the patients’ demographic information and chronic comorbidities that could trigger a depression when diagnosed with AS. By comparing the cohort data, the hazard ratio of developing subsequent depression in AS patients was calculated and adjusted based on several risk factors. Despite the adjustment of demographic variables and chronic comorbidities, the risk of depression was 2.21 times higher in the AS cohort than in the control group. Multivariate analysis showed that AS patients with female gender, old age and low-income status showed higher risks of developing depression. Additionally, the presence of chronic comorbidities including diabetes mellitus, hypertension, hyperlipidemia, cancer, stroke, and chronic kidney disease increased the patients’ risk of depression. The AS patients with stroke were reported to have the highest risk of depression. This population-based cohort study showed that AS significantly increased the subsequent risk of developing depression. Moreover, the development of a depression is influenced by certain demographic variables and different chronic comorbidities.

## Introduction

Ankylosing spondylitis (AS) is a chronic inflammatory rheumatic disease that mainly affects the axial skeleton, resulting in structural and functional impairments^1^. The main symptoms of AS patients include musculoskeletal pain, stiffness, and fatigue^2^. In addition, the first wave of symptoms usually starts before 30 years, and the progressive nature of the disease affects the physical function, causing deterioration of the patients’ professional capacity and quality of life^3^. AS is irreversible and requires lifelong treatments. This poses an increased risk of developing psychological disorders such as depression^4^. Compared with rheumatoid arthritis, the nationwide longitudinal follow-up data of depression prevalence among AS patients is scarce in its size and depth.

Numerous studies have already shown that the prevalence of depression is higher among the patients with chronic disease and that the psychological aspect of treatment is as critical as the clinical treatment^5–7^. Recent studies reveal that patients with patients with AS have an increased risk of developing chronic diseases such as diabetes mellitus and cardiovascular and cerebrovascular diseases^8–11^. Therefore, in the clinical approach to AS patients, it is essential to determine any other existing chronic diseases and be aware of the risks of developing depression from such diseases.

This study aims to have reviewed the prevalence of diagnosing depression in AS patients versus the general population using a nationwide population-based cohort study. Furthermore, our team compared the prevalence of chronic disease in AS patients to the general population based on the Korean National Health Insurance System (KNHIS) data and examined varying risks of developing depression in patients with existing chronic diseases.

## Methods

### Data source

This study used a nationwide dataset available in the Korean National Health Insurance System (KNHIS) collected from 2010 to 2014. In South Korea, the government has implemented an obligatory National Health Insurance (NHI) system, covering 97% of the population and allowing patients to pay only about 30% of the total healthcare cost. The remaining 3% of the population is the lowest income households, and the Medical Aid Program covers all their medical expenses. Healthcare institutions submit claims for the remaining 70% of the total medical cost to the government. Therefore, the medical information of almost all patients in healthcare institutions are prospectively integrated into the KNHIS claim database. The KNHIS claim database includes extensive information on the diagnoses and comorbidity codes classified by the International Classification of Diseases, 10^th^ revision (ICD-10), demographic characteristics, admission and ambulatory care, prescription records, and procedure codes.

### Data collection

We filtered the ICD-10 codes to identify all patients with AS who were included in the KNHIS database from January 2010 to December 2014. The ICD-10 codes for AS (M45.0–45.9) in the Rare Intractable Diseases (RIDs) program were used. The NHI has included AS in the Rare Intractable Diseases (RIDs) registration program since 2009. In the RID grogram, patients were diagnosed with AS if they met the modified New York criteria^12^. The NHI reviews the RID program application again to ensure the diagnostic criteria are met. (Because patients with an RID receive more coverage and pay only about 10% of the total medical cost) This process ensures that diagnoses for the RID program are reliable^13^.

After AS diagnosis, the following categories were used to find demographic variables and comorbidity rate. The low-income household group was defined as the bottom quintile of the general population. Urban resident group were stratified from cities consisting of more than 1 million population (Seoul, Busan, Incheon, Daegu, Daejeon, Ulsan, and Gwangju).

Using ICD-10 codes, the selected baseline comorbidities included diabetes mellitus (ICD-10, E11-14), hypertension (ICD-10, I10-13,15), hyperlipidemia (ICD-10, E78), and stroke (ICD-10, I63, 64). Comorbidities were recognized if the disease appeared in subjects prompted three or more visits in the principal and/or secondary diagnose. To increase the diagnostic code validity, the suitability of prescription drugs was reviewed for selected diseases.

Patients with cancer were selected from patients diagnosed with ICD-10 codes beginning with “C” and registered in the government’s special medical subsidy program. Likewise, patients with end-stage renal disease (ESRD) were chosen from patients who had ICD-10 codes N18 or N19 and underwent renal replacement therapy such as hemodialysis and peritoneal dialysis. Lastly, patients with depression were selected from those who had undergone medical treatment with ICD-10 codes F32 or F33.

### Study design and cohort

**Figure 1:**
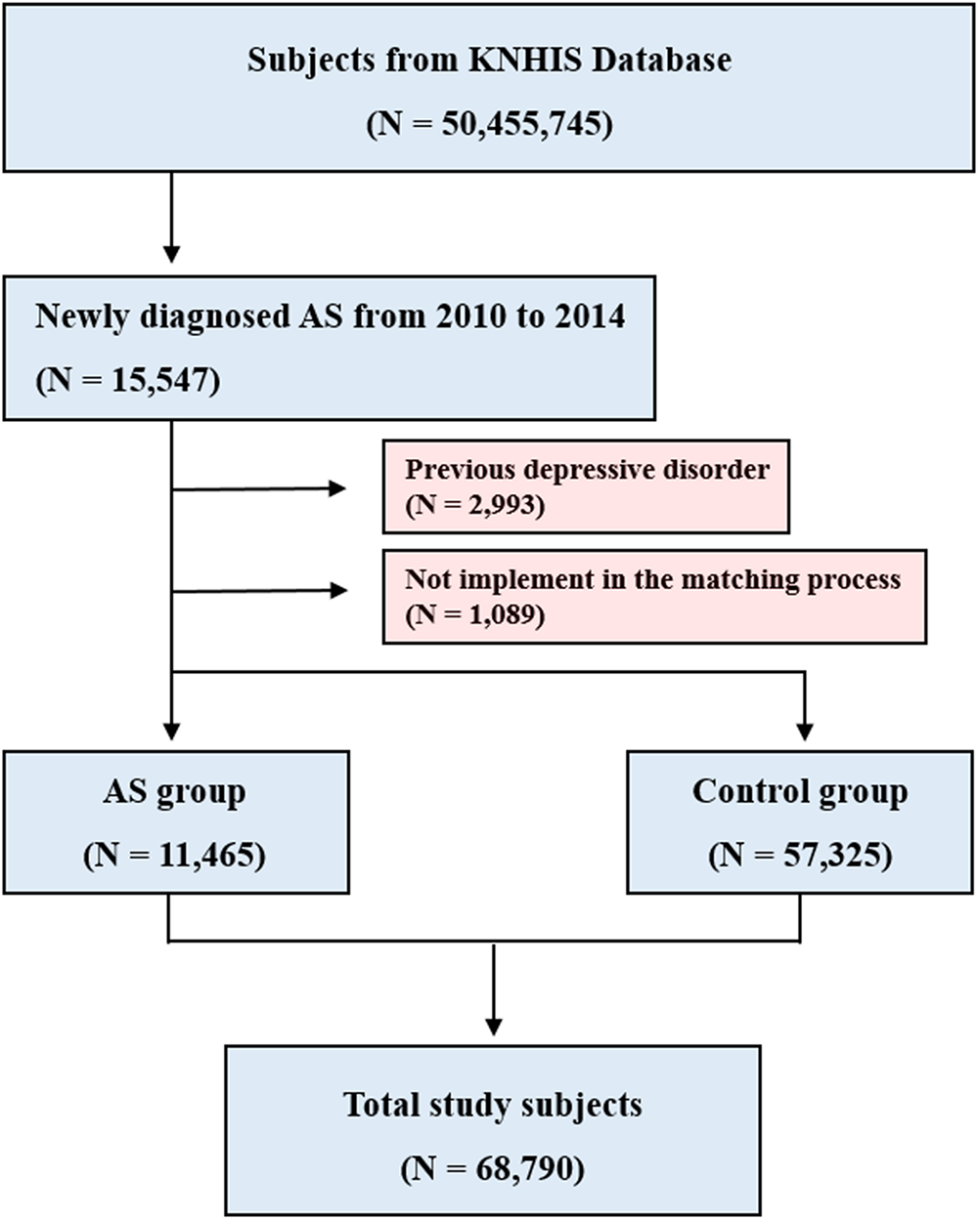
Flowchart of the subjects from the Korean National Health Insurance System (KNHIS).

From the KNHIS database, we identified the AS group which consisted of newly diagnosed AS patients from 2010 to 2014. To firmly support the data on the prevalence of developing depression after diagnosed with AS, 2,993 patients who were diagnosed with depression at least once since 2002 were excluded from the study. Then, the gender and age for newly diagnosed AS patients were matched with the control group in a 1:5 ratio. Patients in the control group were randomly sorted before being selected from top to bottom to prevent selection bias. The group without five control individuals was excluded in the matching process (n = 1,089). The result of our study compared the prevalence of depression among 11,465 newly diagnosed AS patients and 57,325 patients in the control group in December 2015 (Fig. 1).

### Ethical statement

The study protocol was approved by the KNHIS institutional review board. An informed-consent exemption was granted by the board.

### Statistical analysis

The Chi-squared test was used to examine the different demographic variables and disease phenotypes between the AS and control cohorts. The depression-free survival rate and differences between the two groups were evaluated using the Kaplan–Meier method and the log-rank test, respectively. The Cox proportional hazards regression analysis was used to calculate the hazard ratio (HR) and 95% confidence interval (CI) of depression for AS patients compared with the control group. The multivariate Cox proportional hazards model was used to identify the risk factors of depression and adjusted hazard ratio within the AS cohort. Statistical analyses were performed using SAS version 9.3 (SAS Institute Inc., Cary, NC, USA). P-values less than 0.05 were considered statistically significant.

## Results

### Characteristics and depression incidence between the two cohorts

The distributions of demographic variables and chronic disease between AS patients and control cohorts are shown in Table 1. Because of the 1:5 age-sex stratified matching, the proportion of sex and mean age was identical for both groups. On the other hand, AS patients are generally distributed in the low-income household group and living in urban areas. Comorbidities, including diabetes mellitus, hypertension, hyperlipidemia, cancer, stroke, and chronic renal disease, were higher in AS patients (p < 0.001). During the follow-up period, the incidence of newly diagnosed depression was 10.84% (n = 1,243) in the AS group (n = 11,465) and 5.05% (n = 2,894) in the control group (n = 57,325). Hence, the incidence of depression in the AS group was significantly higher than that in the control group (p < 0.001) (Table 1).

**Table 1:** Comparison of demographic characteristics and comorbidities between ankylosing spondylitis (AS) patients and the control group.

	Controls	AS patients	p-value
Number	n = 57325	n = 11465	
Sex (male)	43490 (75.87)	8698 (75.87)	1
Age	40.04 ± 14.51	40.04 ± 14.52	1
≥40 years	25495 (44.47)	5099 (44.47)	1
Low-income status	12522 (21.84)	2705 (23.59)	<0.0001
Residence (urban)	26601 (46.54)	5617 (49.57)	<0.0001
Diabetes	2364 (4.12)	564 (4.92)	0.0001
Hypertension	5873 (10.25)	1613 (14.07)	<0.0001
Hyperlipidemia	3584 (6.25)	1020 (8.9)	<0.0001
Cancer	694 (1.21)	202 (1.76)	<0.0001
Stroke	506 (0.88)	174 (1.52)	<0.0001
End-stage renal disease	64 (0.11)	44 (0.38)	<0.0001
Depression	2894 (5.05)	1243 (10.84)	<0.0001

### Risk of depression in AS patients

Among 68,790 patients (11,465 AS patients and 57,325 control subjects), a total of 4,137 individuals developed depression (1,243 AS patients and 2,894 control subjects during the follow-up of 37,275.35 and 195,703.29 person-years, respectively.) The incidence rate of depression in the AS cohort was greater than that in the control group (33.35 versus 14.79 per 1,000 person-years).

**Figure 2:**
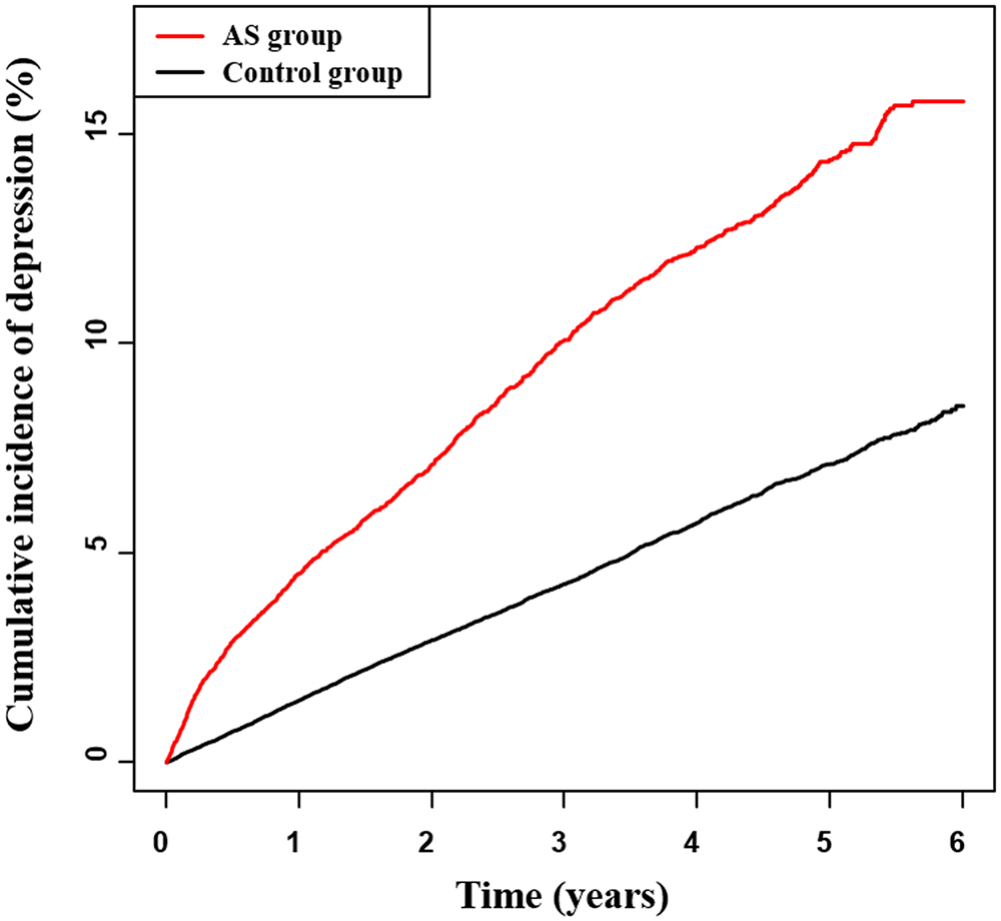
The Kaplan–Meier curves showed significantly higher cumulative incidence of overall depression in ankylosing spondylitis (AS) patients than those in the non-AS control group (p < 0.001).

The multivariate Cox proportional hazards regression analysis was used to estimate the HR of newly diagnosed depression among AS patients based on the statistical adjustment (Model 1: age and sex, Model 2: age, sex, household income, diabetes mellitus, hypertension, hyperlipidemia, stroke, cancer, and ESRD). A markedly higher risk of developing subsequent depression was noted in AS patients than in the matched controls (adjusted HR: 2.3, 95% CI: 2.152–2.458 in Model 1 and adjusted HR: 2.211, 95% CI: 2.068–2.363 in Model 2) (Table 2). The KM curves with cumulative hazards showed a significantly higher incidence of overall depression in the AS group than the control group (p-value for the log-rank test <0.001). Moreover, the gap difference between two KM curves of the AS and control groups increased with time (Fig. 2).

**Table 2:** Crude and adjusted hazard ratios of depression in ankylosing spondylitis (AS) patients compared with the non-AS control group.

Patient group	N	Depression	Duration	Rate	HR (95% CI)	
					Crude HR^a^	Adjusted HR^b^
Controls	57325	2894	195703.29	14.788	1 (ref.)	1 (ref.)
AS patients	11465	1243	37275.35	33.346	2.3 (2.152,2.458)	2.211 (2.068,2.363)

Crude HR^a^; age, sex.

Adjusted HR^b^; age, sex, income, residence, diabetes mellitus, Hypertension, Hyperlipidemia, stroke, cancer, end-stage renal disease.

### Subgroup analysis of the risk of depression in AS patients

The multivariate Cox proportional hazard regression analysis was used to estimate the adjusted HR of depression among AS patients based on the demographic data and chronic disease comorbidity (Table 3). Compared to the male gender, the female gender had a higher risk of depression in AS patients (HR: 1.60, 95% CI: 1.50–1.71). Higher depression rate was observed in patients diagnosed with AS above 40 years than those diagnosed below 40 years (HR: 1.78, 95% CI: 1.65–1.90). The low-income group (bottom 20% of the general income) appeared to have a mildly higher risk of developing depression (HR: 1.28, 95% CI: 1.20–1.37). There was little difference in the risk of depression based on residence (HR: 1.08, 95% CI: 1.01–1.14). Additionally, the presence of comorbidities increased the risk of depression. AS patients with stroke have the highest risk (HR: 2.06, 95% CI: 1.73–2.43), followed by those with ESRD (HR: 2.05, 95% CI: 1.35–3.09), with cancer (HR: 1.65, 95% CI: 1.37–1.99), with hypertension (HR: 1.49, 95% CI: 1.36–1.63), with dyslipidemia (HR: 1.32, 95% CI: 1.19–1.47), and with diabetes mellitus (HR: 1.26, 95% CI: 1.12–1.41) (Table 3).

**Table 3:** Multivariate analysis of factors related to the risk of depression in ankylosing spondylitis patients

Variables		Crude HR (95% CI)	Adjusted HR^a^
Sex	Male	1 (ref.)	1 (ref.)
	Female	1.744 (1.637,1.859)	1.602 (1.502,1.708)
Age group	<40 years	1 (ref.)	1 (ref.)
	≥40 years	2.273 (2.133,2.422)	1.771 (1.652,1.898)
Income	Other	1 (ref.)	1 (ref.)
	Low 20%	1.373 (1.283,1.47)	1.281 (1.197,1.372)
Residence	Urban	1 (ref.)	1 (ref.)
	Rural	1.075 (1.011,1.144)	1.075 (1.01,1.143)
Diabetes	No	1 (ref.)	1 (ref.)
	Yes	2.418 (2.175,2.688)	1.258 (1.118,1.415)
Hypertension	No	1 (ref.)	1 (ref.)
	Yes	2.523 (2.346,2.714)	1.489 (1.363,1.627)
Hyperlipidemia	No	1 (ref.)	1 (ref.)
	Yes	2.475 (2.268,2.702)	1.323 (1.194,1.467)
Cancer	No	1 (ref.)	1 (ref.)
	Yes	2.478 (2.059,2.983)	1.648 (1.367,1.987)
Stroke	No	1 (ref.)	1 (ref.)
	Yes	4.159 (3.53,4.9)	2.057 (1.734,2.439)
End-stage renal disease	No	1 (ref.)	1 (ref.)
	Yes	3.978 (2.641,5.993)	2.045 (1.354,3.089)

Adjusted HR^a^; adjusted for all variables in the mode.

## Discussion

This is a cohort study that determines the risk of subsequent depression following the diagnosis of AS based on the national administrative data. This study has shown that AS patients had higher prevalence of chronic comorbidities (diabetes mellitus, hypertension, hyperlipidemia, cancer, stroke, ESRD) compared to the control group in terms of the age and sex. In addition, the income level and location of residence affects the prevalence of AS. Even after negating the effects of demographic variables and comorbidity, the study has revealed that AS patients are 2.21 times more likely to develop the risk of depression than those without AS.

Numerous studies involving the relationship between AS and depression have been conducted^2,4,6,14^. In AS patients, inflammatory pain caused sleep disturbance and lowered the fatigue level in social life. In other words, the severity of the disease affects the individual’s quality of life and increases the risk of depression unless the disease activity in AS has fully subsided^2,3,15^. Similarly, AS patients exhibited poor functional outcome at work, and their consequent socioeconomic status influenced the prevalence of depression^16,17^. Recent studies reported that proinflammatory cytokines take important roles in the pathophysiology of depression^18^. In other words, systemic inflammation and proinflammatory cytokines of AS are associated with depression^19^. This is also supported by the fact that infliximab (tumor necrosis factor-α drug) is effective in the treatment of AS patients with depression^20^. Hence, the biological, functional, and socioeconomic factors of AS patients increase the risk of depression.

However, few studies have examined the data on subsequent depression in AS patients compared to those with rheumatoid arthritis. In the previous AS cohort study consisting 1,738 out of 967,012 subjects, 10% of the AS cohort had a doctor-diagnosed depression compared to 6% of the control group in the 13-year observational period. Furthermore, the risk of depression was higher in females and in AS patients with diabetes mellitus^4^. However, person-time for residents not seeking healthcare during the study time frame was not included, and any other chronic comorbidities other than diabetes mellitus were not considered.

Studies have shown that the prevalence of comorbidities including cardiovascular disease, stroke, diabetes mellitus, and renal disease is higher in AS patients^10,11,21,22^. The direct cause has not yet been established; however, it is speculated to be the common pathological pathway or biological background of the disease. Several studies have already presented the importance of the psychological aspect such as depression in the treatment of patients with chronic diseases. Additionally, recent studies show that drugs for chronic diseases, such as oncologic medication, corticosteroids, or biological agents, can trigger depressive episodes. Therefore, it is important to identify any chronic comorbidities in AS patients to factor in the risk of depression from those comorbidities.

This study explains the temporal association between AS and the prevalence of depression. Compared to the control group, AS patients had greater risk of depression as the time progressed from the initial diagnosis. In the fifth year following the initial diagnosis, 1.5 out of 10 AS patients and 0.5 out of 10 non-AS patients required the administration of anti-depressant drugs. Females and low-income patients were more susceptible to depression. Additionally, patients above 40 years who were diagnosed with AS were more vulnerable to depression than those below 40 years who were aware of their condition. This study examined the risk of developing depression in various comorbidities including stroke, cancer, chronic kidney disease, hypertension, diabetes mellitus, and hyperlipidemia, with the stroke comorbidity being the highest. These results, altogether, will provide substantive and applicable information of the psychological aspect in the treatment of AS patients.

There are certain limitations of our study. First, the KNHIS dataset does not contain detailed information on the physical activities, body mass index, or history of smoking, alcohol consumption, and family history of psychological disorders; all of which may be associated with psychological disorders. Although our team was not able to adjust for all potential confounding variables, the study is noteworthy for including different comorbidities and varying demographic characteristics, residential locality, and household income and proves that AS patients have significantly higher risk of developing depression. Second, miscoding of ICD-10 diagnostic code could have been done. Nevertheless, this study rechecks the appropriateness of prescription drugs to minimize error and maximize reliability of the diagnostic code. Third, because we had to use administrative data, the severity and duration of symptoms were not considered in this study. This study focused on the risk of developing depression after the point of AS diagnosis.

In conclusion, this study has shown that patients with AS is 2.21 times more likely to develop the risk of depression than the general population, even after negating several potentially influential variables of demographic characteristics and comorbid conditions. Our population-based study has also revealed that AS is more common among low-income individuals with other chronic diseases. Clinicians treating AS patients should acknowledge the demographic characteristics, comorbidity, risk factors, and latent risk for depression.
